# A Feynman Path Integral-like Method for Deriving Reaction–Diffusion Equations

**DOI:** 10.3390/polym14235156

**Published:** 2022-11-27

**Authors:** Changhao Li, Jianfeng Li, Yuliang Yang

**Affiliations:** The State Key Laboratory of Molecular Engineering of Polymers, Department of Macromolecular Science, Fudan University, Shanghai 200433, China

**Keywords:** reaction–diffusion equation, path integral

## Abstract

This work is devoted to deriving a more accurate reaction–diffusion equation for an A/B binary system by summing over microscopic trajectories. By noting that an originally simple physical trajectory might be much more complicated when the reactions are incorporated, we introduce diffusion–reaction–diffusion (DRD) diagrams, similar to the Feynman diagram, to derive the equation. It is found that when there is no intermolecular interaction between A and B, the newly derived equation is reduced to the classical reaction–diffusion equation. However, when there is intermolecular interaction, the newly derived equation shows that there are coupling terms between the diffusion and the reaction, which will be manifested on the mesoscopic scale. The DRD diagram method can be also applied to derive a more accurate dynamical equation for the description of chemical reactions occurred in polymeric systems, such as polymerizations, since the diffusion and the reaction may couple more deeply than that of small molecules.

## 1. Introduction

Finding correct and thermodynamically consistent reaction–diffusion equations [1,2,3,4,5] is important for the study of both small-molecule and macromolecular reacting systems. Previously, the reaction–diffusion equations were obtained by simply adding the contributions from the reaction and the diffusion together [1,2,3,4,5], and they were, thus, assumed to be independent of each other.

However, experiments [6,7] revealed that diffusions and chemical reactions may affect each other, and these coupling effects will cause observable consequences. For example, Wang et al. [6] found that chemical reactions will change the mobility of molecules or affect diffusions by kicking the molecules near the reaction sites. Accordingly, diffusions can also affect chemical reactions. Liu et al.’s recent work [7] showed that because in the polymerization, monomers have to be transported, by passing through a complicated entanglement of the long chain to the right location of the reaction site of a polymer chain in order for the chain growth reaction to actually happen, a wait-and-jump phenomenon was observed in the chain growth polymerization.

Therefore, it is necessary to propose a theoretical framework that can be used to describe the coupling between chemical reactions and diffusions. The common framework used to investigate this coupling is the diffusion-controlled reaction model. For example, based on the formulation of second quantization or the path integral, Doi and Peliti [8,9,10] successively proposed a very general theoretical framework for the dynamics of many-particle system, which can, in principle, be used to derived the dynamical equation for describing the reaction–diffusion coupling. However, as pointed out by Doi himself, the second-quantization method might not be very useful for the usual classical systems. Another method is the Smoluchowski diffusion limited reaction model [11,12], which is mainly based on the Smoluchowski equation of this system. Recently, the reaction–diffusion master equation (RDME) model has also been attracting the attention of researchers for stochastic chemical kinetics [13,14,15,16], where the particles are treated as points and evolve according to master equations.

However, these models studied diffusion–reaction coupling by investigating bimolecular reactions. One may, therefore, be interested to know whether this coupling still exists in monomolecular reactions. Furthermore, if this coupling exists in the monomolecular case, can we simply add the contributions from the reaction and diffusion together to derive dynamic equations? Additionally, to what extent can we neglect the coupling term?

Therefore, this work is devoted to re-deriving the reaction–diffusion equation by summing over the contributions from all possible trajectories of reactions and diffusions. Hopefully, we can identify the coupling terms of these two processes in the newly derived reaction–diffusion equation.

**Figure 1:**
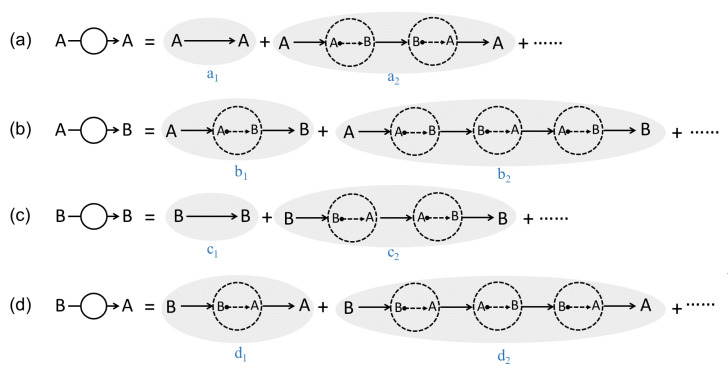
Diffusion–reaction–diffusion (DRD) diagrams of four processes related with the A/B binary reaction–diffusion system. In the diagram, the solid arrow indicates diffusion, and the dotted arrow means chemical reaction. (**a**,**c**) The diffusions of A or B component from r to r+Δr within a given amount of time Δτ actually consists of a bunch of possible DRD diagrams in the A/B reaction system. The reactions A→B (**b**) and B→A (**d**) also consist of numerous DRD diagrams. Note that a given diagram actually corresponds to an ensemble of trajectories.

Inspired by the Feynman diagram in particle physics, this work will introduce a series of diffusion–reaction–diffusion (DRD) diagrams to derive a more accurate reaction–diffusion equation. The basic idea of the DRD diagram, taking a binary reacting system (A⇌B) as an example, is as follows. Previously, most of us might see a trajectory (or more precisely, a trajectory ensemble) of an A-type molecule moving from r to another point $r^{\prime }$ at Δτ later as just one physical process denoted as $\left(A;r,t\right)\to \left(A;r^{\prime },t+\Delta \tau \right)$, and summing all possible $r^{\prime }$ and Δτ will lead to the diffusion equation of A. This strategy has been employed by many previous works [17,18,19] to derive field-based dynamical equations based on the particle-based microscopic dynamics. However, this work notices that when A can turn into B and B can turn into A through chemical reactions, in between the trajectory of A→A, other physical processes can also occur. For example, even though the physical process (see $a_{2}$ in Figure 1a) $\left(A;r,t\right)\to \left(A;r^{\prime \prime },t+\Delta \tau^{\prime }\right)⤏\left(B;r^{\prime \prime },t+\Delta \tau^{\prime }\right)\to \left(B;r^{\prime \prime \prime },t+\Delta \tau^{\prime \prime }\right)⤏\left(A;r^{\prime \prime \prime },t+\Delta \tau^{\prime \prime }\right)\to \left(A;r^{\prime },t+\Delta \tau \right)$$\left(A;r,t\right)\to \left(A;r^{\prime },t+\Delta \tau \right)$ involves two chemical reaction processes in between r and $r^{\prime }$, it can be still seen as the above process $\left(A;r,t\right)\to \left(A;r^{\prime },t+\Delta \tau \right)$ by us. Therefore, a simple physical process $\left(A;r,t\right)\to \left(A;r^{\prime },t+\Delta \tau \right)$ might have many possible variants. This work is going to consider these possibilities, and we hope to derive a more accurate reaction–diffusion equation based on this idea. In particular, we are specially interested in what conditions the reaction will couple with diffusions according to the newly derived equations, which might also have potential applications for the description of the dynamics in the mesoscopic scale or the dynamics of polymerization.

## 2. Diffusion–Reaction–Diffusion Diagram

In this section, we will give a brief introduction about the overall strategy on how to derive the dynamical equation of a binary system undergoing chemical reactions and diffusions. In particular, the diffusion–reaction–diffusion (DRD) diagram, which is similar to the Feynman diagram in particle physics, will be specially introduced.

The overall derivation strategy is simple and direct. First, calculate the probabilities of all possible physical processes or trajectories. Second, evaluate the contributions of these trajectories to the number density variation based on their probabilities. Third, obtain the number density variation (or dynamical equation) of a given component by summing over the contributions from all possible trajectories.

In the present binary system undergoing chemical reactions, there are mainly four physical processes as shown in Figure 1, (a) diffusion of an A molecule from r to r+Δr within a small time span Δτ, (b) an A-type molecule turns into a B-type molecule through chemical reaction, (c) diffusion of a B molecule, and (d) a B-type molecule turns into an A-type molecule. Just as pointed out in the introduction section, each of these physical processes is not just a single process but actually represents a group of physical processes.

For example, for the first group physical processes (a), one might observe an *A* molecule disappearing at r at t=0 and re-appearing at r+Δr at t=Δτ. This phenomenon may correspond to many possible physical processes as shown in Figure 1, where the A molecule can turn into B molecule through chemical reaction. Take the $a_{2}$ DRD diagram for example (Figure 1a); the A molecule first diffuses into another position and turns into B at that position, which will further diffuse into somewhere, turn back into A, and finally diffuse to the position r+Δr with the whole process taking a time of Δτ. Obviously, in between A→A diffusion process, one can add as many (A⤏B)+(B⤏A) chemical reaction pairs as possible as long as the whole process takes the time of Δτ and the final position is r+Δr.

**Figure 2:**
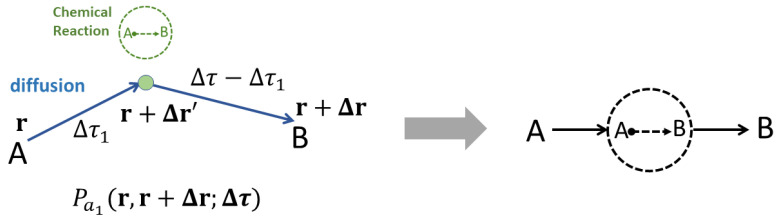
The left panel shows more information about the $b_{1}$ diagram of Figure 1b, which indicates that in this process, an A molecule will firstly diffuse from r to $r+\Delta r^{\prime }$ within $\Delta \tau_{1}$, then turn into the B molecule through chemical reaction (green dot) at the position $r+\Delta r^{\prime }$, and finally B diffuses to the position r+Δr within $\Delta \tau -\Delta \tau_{1}$. The probability of this process is denoted as $P_{b_{1}}\left(r,r+\Delta r;\Delta \tau \right)$, which is obtained by path integrating over all possible trajectories $r\to r+\Delta r^{\prime }$ and $r+\Delta r^{\prime }\to r+\Delta r$ and by integrating over the intermediate variables $\Delta r^{\prime }$ and $\Delta \tau_{1}$.

More information about these DRD diagrams can be seen in Figure 2.

If the probabilities $P_{x_{i}}\left(r,r+\Delta r;\Delta \tau \right)$ (x={a,b,c,d} and i=1,2,3…) of these DRD diagrams can be calculated, then the probabilities of these four groups of processes can be obtained as $P_{x}\left(r,r+\Delta r;\Delta \tau \right)=\sum_{i}P_{x_{i}}\left(r,r+\Delta r;\Delta \tau \right)$. Luckily, the order of $P_{x_{i}}$ in Δτ is completely determined by how many chemical reactions occur in between the process (e.g., $P_{a_{1}}∼\Delta \tau^{0}$ and $P_{b_{2}}∼\Delta \tau^{3}$), then most of the DRD diagrams of a higher order can be neglected if Δτ is small.

With $P_{x_{i}}$’s, the dynamical equation of A can be derived as follows:

(1) $$\begin{array}{ccc} \frac{\partial \phi_{A}\left(r\right)}{\partial t}= & & \lim_{\Delta \tau \to 0}\sum_{i}\int d\Delta r[\phi_{A}\left(r+\Delta r\right)P_{a_{i}}\left(r+\Delta r,r;\Delta \tau \right)-\phi_{A}\left(r\right)P_{a_{i}}\left(r,r+\Delta r;\Delta \tau \right)\\ & & +\phi_{B}\left(r+\Delta r\right)P_{d_{i}}\left(r+\Delta r,r;\Delta \tau \right)-\phi_{A}\left(r\right)P_{b_{i}}\left(r,r+\Delta r;\Delta \tau \right)], \end{array}$$

where $\phi_{A/B}$ are the number densities of A and B, respectively. Note that this equation is similar to the master equation in Doi’s work [9]. The equation for B is similar. We shall explain the four terms on the right hand side of the above equation one by one as follow. The first two terms account for the diffusions of A from r to r+Δr and from r+Δr to r, respectively. The third term $\phi_{B}\left(r+\Delta r\right)P_{d_{i}}\left(r+\Delta r,r;\Delta \tau \right)$ accounts for the density increase due to the chemical reaction B⤏A and it is obviously proportional to number of B molecules found at r+Δr. The last term is related to the reaction A⤏B.

Evaluations of these four terms are quite involved and will be presented in Section 3, Section 4 and Section 5.

## 3. DRD Diagram Rules

If A/B molecules are assumed to obey Langevin dynamics, it can be proved that the probability of a pure diffusion DRD diagram ($a_{1}$ and $c_{1}$ in Figure 1) can be obtained as follows, by path integrating all possible trajectories connecting r and r+Δr (see Equation (A7) in Appendix B):

(2) $$\begin{array}{c} P_{A}\left(r,r+\Delta r;\Delta \tau \right)={\left(\frac{1}{4\pi D\Delta \tau }\right)}^{\frac{3}{2}}e^{-\frac{\Delta r^{2}}{4D\Delta \tau }}e^{\frac{1}{2}\left[U_{A}\left(r\right)-U_{A}\left(r+\Delta r\right)\right]}, \end{array}$$

where *D* is the diffusion coefficient. $U_{A}\left(r\right)$ is the potential field felt by a single molecule A at r. For example, when there is interaction $\chi \phi_{A}\phi_{B}$ between A and B, then $U_{A}\left(r\right)=\chi \phi_{B}^{2}$. Note that chemical potential of A can be expressed as $\mu_{A}=\ln\phi_{A}+U_{A}+\epsilon_{A}$ with $\mu_{A}^{0}=\epsilon_{A}$ the inner energy of A. Δτ is chosen such that it should be larger than the minimum time scale that makes Langevin dynamics valid for A and B molecules. Note that, for the sake of simplicity, we set $k_{B}T=1$ throughout this work.

Similarly, for the pure diffusion of B, we have

(3) $$\begin{array}{c} P_{B}\left(r,r+\Delta r;\Delta \tau \right)={\left(\frac{1}{4\pi D\Delta \tau }\right)}^{\frac{3}{2}}e^{-\frac{\Delta r^{2}}{4D\Delta \tau }}e^{\frac{1}{2}\left[U_{B}\left(r\right)-U_{B}\left(r+\Delta r\right)\right]}. \end{array}$$

With Equations (2) and (3), we have the following **diagram rules** (similar to the Feynman rules):

(i) The two end points of the DRD diagram can be either at r or r+Δr.
(ii) Each internal vertex (dotted circle) represents a chemical reaction, either A⤏B or B⤏A, but it should be consistent with its incoming or outgoing molecule. For example, if the incoming molecule is A, then the reaction should be A⤏B (we use the dash arrow to denote the reaction).
(iii) A⤏B vertex contributes a factor $k^{+}\exp\left\{U_{A}\left(r+\Delta r^{\prime }\right)\right\}$ with $r+\Delta r^{\prime }$ the spatial position of the vertex, while B⤏A vertex contributes a factor $k^{-}\exp\left\{U_{B}\left(r+\Delta r^{\prime }\right)\right\}$ with $k^{+}$ and $k^{-}$, the reaction rate constants of the forward and the reverse reactions, respectively.
(iv) A solid-line arrow represents a pure diffusion process, and it contributes a factor $P_{A/B}\left(r_{i},r_{i+1};\Delta \tau_{i}\right)$ as given in Equations (2) and (3), where $r_{i}$ and $r_{i+1}$ denotes the spatial positions of the two end points of the arrow, and $\Delta \tau_{i}$ denotes the time span of the diffusion. Note that $\sum_{i}\Delta \tau_{i}=\Delta \tau$ and $\Delta \tau_{i}>0$.
(v) Multiplying all the factors of arrows ($P_{A/B}$) and vertices ($k^{\pm }\exp\left\{U_{A/B}\left(r+\Delta r^{\prime }\right)\right\}$) and integrating over all possible spatial positions of vertices and time spans of the arrows, the probability of the diagram can be finally evaluated.

Comments about (iii). Note that even though we have not explicitly let the probability of reaction depend on the time span, when being incorporated with the diffusion process, the reaction DRD diagram will be found to depend on the time span (see Equation (6)). The forward and backward reaction rates are related by $k^{+}=k_{0}e^{\epsilon_{A}}$ and $k^{-}=k_{0}e^{\epsilon_{B}}$ with $\epsilon_{A}=\mu_{A0}$ and $\epsilon_{B}=\mu_{B0}$, the inner energies of A and B, respectively.

The reason we choose the factor (the local reaction probability) of the reaction vertex of A⤏B as $k^{+}\exp\left\{U_{A}\left(r+\Delta r^{\prime }\right)\right\}$ is as follows. First, the probability of the forward reaction of A⤏B should locally depend on only the state of the reactant A other than that of the product B. Physically, A’s state is totally determined by its inner energy $\epsilon_{A}$ and the external field felt $U_{A}$ by A, so the probability function of A⤏B should take the form of $f(\epsilon_{A},U_{A})$. The expression of $k^{+}=k_{0}e^{\epsilon_{A}}$ obviously indicates $f\left(\epsilon_{A},U_{A}\right)=e^{\epsilon_{A}}g\left(U_{A}\right)$, and it is natural to conjecture $g\left(U_{A}\right)=e^{U_{A}}$. Second, as can be seen in Section 6 and Section 7, the derived reaction–diffusion equation using this factor is more reasonable than those obtained using other methods, especially for the small density region.

## 4. Probability of DRD Diagram

### 4.1. $b_{1}$ Diagram’s Probability

For example, based on the above five rules, we can evaluate the DRD diagram of $b_{1}$ (Figure 2) as follows:

(4) $$\begin{array}{ccc} P_{b_{1}}\left(r,r+\Delta r;\Delta \tau \right) & = & \int \int d\Delta r^{\prime }d\Delta \tau^{\prime }P_{A}\left(r,r+\Delta r^{\prime };\Delta \tau^{\prime }\right)k^{+}\exp\left[U_{A}\left(r+\Delta r^{\prime }\right)\right]\\ & \times & P_{B}\left(r+\Delta r^{\prime },r+\Delta r;\Delta \tau -\Delta \tau^{\prime }\right). \end{array}$$

Plugging Equations (2) and (3) into the above equation and re-arranging the relevant terms, the above equation can be rewritten as

(5) $$\begin{array}{ccc} & & P_{b_{1}}\left(r,r+\Delta r;\Delta \tau \right)=f\left(r\right)h\left(r+\Delta r\right)\int \int d\Delta rd\tau^{\prime }{\left(\frac{1}{4D\pi \tau^{\prime }}\right)}^{\frac{3}{2}}{\left[\frac{1}{4D\pi (\tau -\tau^{\prime })}\right]}^{\frac{3}{2}}\\ & & \times g\left(r+\Delta r^{\prime }\right)\exp\left[-\frac{\Delta r^{\prime 2}}{4D\tau^{\prime }}-\frac{{\left(\Delta r-\Delta r^{\prime }\right)}^{2}}{4D(\tau -\tau^{\prime })}\right], \end{array}$$

where

$$\begin{array}{ccc} & & f\left(r\right)=k^{+}\exp\left\{\frac{1}{2}U_{A}\left(r\right)\right\}\\ & & h\left(r+\Delta r\right)=\exp[-\frac{1}{2}U_{B}\left(r+\Delta r\right)]\\ & & g\left(r+\Delta r^{\prime }\right)=\exp[\frac{1}{2}\left[U_{A}\left(r+\Delta r^{\prime }\right)+U_{B}\left(r+\Delta r^{\prime }\right)\right]. \end{array}$$

By Equation (A5) in Appendix A, $P_{b_{1}}$ can be evaluated as

(6) $$\begin{array}{ccc} & & P_{b_{1}}\left(r,r+\Delta r;\Delta \tau \right)={\left(\frac{1}{4\pi D\Delta \tau }\right)}^{\frac{3}{2}}e^{-\frac{\Delta r^{2}}{4D\Delta \tau }}k^{+}e^{\frac{1}{2}\left[U_{A}-U_{B}\left(r+\Delta r\right)\right]}\{e^{\frac{1}{2}\left(U_{A}+U_{B}\right)}\Delta \tau \\ & & +\frac{1}{2}\left[\nabla e^{\frac{1}{2}\left(U_{A}+U_{B}\right)}\right]\cdot \Delta r\Delta \tau +\frac{1}{6}\left[\partial_{\alpha }\partial_{\beta }e^{\frac{1}{2}\left(U_{A}+U_{B}\right)}\Delta x^{\alpha }\Delta x^{\beta }\Delta \tau +\nabla^{2}e^{\frac{1}{2}\left(U_{A}+U_{B}\right)}D\Delta \tau^{2}+\ldots \right]\}, \end{array}$$

where only terms up to the second order of Δτ are kept, and the meaning of $\partial_{\alpha }\partial_{\beta }g\Delta x^{\alpha }\Delta x^{\beta }$ is referred to the context around Equation (A5).

The probability of the $d_{1}$ diagram (Figure 1d) can be easily obtained by simply switching A and B and replacing $k^{+}$ with $k^{-}$ in Equation (6),:

(7) $$\begin{array}{ccc} & & P_{d_{1}}\left(r,r+\Delta r;\Delta \tau \right)={\left(\frac{1}{4\pi D\Delta \tau }\right)}^{3/2}e^{-\frac{\Delta r^{2}}{4D\Delta \tau }}k^{-}e^{\frac{1}{2}\left[U_{B}-U_{A}\left(r+\Delta r\right)\right]}\{e^{\frac{1}{2}\left(U_{A}+U_{B}\right)}\Delta \tau \\ & & +\frac{1}{2}\left[\nabla e^{\frac{1}{2}\left(U_{A}+U_{B}\right)}\right]\cdot \Delta r\Delta \tau +\frac{1}{6}\left[\partial_{\alpha }\partial_{\beta }e^{\frac{1}{2}\left(U_{A}+U_{B}\right)}\Delta x^{\alpha }\Delta x^{\beta }\Delta \tau +\nabla^{2}e^{\frac{1}{2}\left(U_{A}+U_{B}\right)}D\Delta \tau^{2}+\ldots \right]\}. \end{array}$$

### 4.2. Probability of More Complex Diagrams

After the $b_{1}$ and $d_{1}$ diagrams’ probabilities are obtained, other diagrams’ probabilities can be derived one by one.

For example, $a_{2}$ diagram can be obtained by adding a B⤏A reaction and an A-type diffusion to the $b_{1}$ diagram (see Figure 1a,b). Accordingly, by the DRD diagram’s rule, it can be realized by multiplying $b_{1}$ diagram’s probability $P_{b_{1}}\left(r,r+\Delta r^{\prime };\Delta \tau^{\prime }\right)$ with $k^{-}e^{U_{B}\left(r+\Delta r^{\prime }\right)}$ (B⤏A reaction) and $P_{A}\left(r+\Delta r^{\prime },r+\Delta r;\Delta \tau -\Delta \tau^{\prime }\right)$ (A-type diffusion) and integrating over $\Delta r^{\prime }$ and $\Delta \tau^{\prime }$. Closely following Equation (4), the probability of $a_{2}$ diagram can be written as

(8) $$\begin{array}{ccc} P_{a_{2}}\left(r,r+\Delta r;\Delta \tau \right)= & & \int \int d\Delta r^{\prime }d\Delta \tau^{\prime }P_{b_{1}}\left(r,r+\Delta r^{\prime };\Delta \tau^{\prime }\right)k^{-}\exp\left[U_{B}\left(r+\Delta r^{\prime }\right)\right]\\ & & \times P_{A}\left(r+\Delta r^{\prime },r+\Delta r;\Delta \tau -\Delta \tau^{\prime }\right). \end{array}$$

Even without working out the above integration, one can conclude that the leading order of the $a_{2}$ diagram is of the second order of Δτ.

Similarly, multiplying one more forward reaction and a pure B-type diffusion to $a_{2}$ diagram and integrating the corresponding positions and time intervals, one obtains the $b_{2}$ diagram’s probability. All other diagrams can be derived step by step in this way.

### 4.3. Order, Symmetry and Duality of the Diagram

Here, the order of the diagram is defined as the leading order in Δτ of the diagram’s probability. By the above subsection, one can easily prove that the order of the diagram amounts to the number of reactions or the number of dotted circles in the diagram. Accordingly, $a_{i}$ and $c_{i}$ diagrams are of ∼$\Delta \tau^{2i-2}$, while $b_{i}$ and $d_{i}$ diagrams are of ∼$\Delta \tau^{2i-1}$.

For diagrams $a_{i}$ and $c_{i}$ (Figure 1a,c), reversing the directions of all the sub processes results in the same diagram. For example, we will still obtain the $a_{2}$ diagram after reversing the directions of the $a_{2}$ diagram. Therefore, $a_{i}$ and $c_{i}$ are symmetric in this respect.

On the other hand, $b_{i}$ is not symmetric, but is dual to $d_{i}$, i.e., reversing $b_{i}$ will result in $d_{i}$.

The order, symmetry and duality of the diagram are important in the following derivation of the dynamical equation.

## 5. Contribution of DRD Diagram to the Density Variation

We first present the following two general formulae, which can be used to evaluate the contribution of a DRD diagram to the number density variation, as follows:

(9) $$\begin{array}{ccc} & & \Delta \phi_{X}\left(r;P_{X\to Y},\Delta \tau \right)=-\int d\Delta r\phi_{X}\left(r\right)P_{X\to Y}\left(r,r+\Delta r;\Delta \tau \right), \end{array}$$

(10) $$\begin{array}{ccc} & & \Delta \phi_{Y}\left(r;P_{X\to Y},\Delta \tau \right)=\int d\Delta r\phi_{X}\left(r+\Delta r\right)P_{X\to Y}\left(r+\Delta r,r;\Delta \tau \right), \end{array}$$

where the subscript of $P_{X\to Y}$ indicates that the two chemicals at the two ends of the diagram are X and Y. Note that X and Y can be the same species. These two formulae can be applied to all diagrams in Figure 1.

### 5.1. Asymmetric Diagrams’ Contributions

The contribution of the $b_{1}$ diagram to the density variation of A in Equation (1) can be obtained by multiplying the density $\phi_{A}\left(r\right)$ and integrating over Δr, as follows:

(11) $$\begin{array}{ccc} & & \Delta \phi_{A}\left(r;P_{b_{1}},\Delta \tau \right)=-\int d\Delta r\phi_{A}\left(r\right)P_{b_{1}}\left(r,r+\Delta r;\Delta \tau \right)\\ & & =-k_{0}e^{\mu_{A}}\Delta \tau -Dk_{0}e^{\mu_{A}}\Delta \tau^{2}\left[\frac{1}{8}{\left(\nabla U_{A}\right)}^{2}+\frac{1}{8}{\left(\nabla U_{B}\right)}^{2}+\frac{1}{4}\nabla^{2}\Delta U\right]+\ldots \end{array}$$

where the minus sign indicates that the $b_{1}$ diagram causes $\phi_{A}$ to decrease, $\mu_{A}=U_{A}+\epsilon_{A}+\ln\phi_{A}$ and $\Delta U\equiv U_{A}-U_{B}$. The detailed derivation of Equation (11) is referred to in Appendix C.

The $b_{1}$ diagram will also contribute to the variation of $\phi_{B}$ as follows:

(12) $$\begin{array}{ccc} & & \Delta \phi_{B}\left(r;P_{b_{1}},\Delta \tau \right)=\int d\Delta r\phi_{A}\left(r+\Delta r\right)P_{b_{1}}\left(r+\Delta r,r;\Delta \tau \right)\\ & & =k_{0}e^{\mu_{A}}\Delta \tau +Dk_{0}e^{\mu_{A}}\Delta \tau^{2}[\frac{1}{8}{\left(\nabla U_{A}\right)}^{2}+\frac{1}{8}{\left(\nabla U_{B}\right)}^{2}-\frac{1}{4}\nabla^{2}\Delta U+{\left(\nabla \mu_{A}\right)}^{2}+\nabla^{2}\mu_{A}\\ & & -\frac{1}{2}\nabla \mu_{A}\cdot \nabla \Delta U]+\ldots \end{array}$$

where $\Delta U\equiv U_{A}-U_{B}$.

As the dual diagram of $b_{1}$, the $d_{1}$ diagram’s contribution to the concentration variation B will be exactly the same as $b_{1}$’s contribution to A (Equation (11)), except that A needs to be replaced with B, and it can be expressed as follows:

(13) $$\begin{array}{ccc} & & \Delta \phi_{B}\left(r;P_{d_{1}},\Delta \tau \right)=\\ & & -k_{0}e^{\mu_{B}}\Delta \tau -Dk_{0}e^{\mu_{B}}\Delta \tau^{2}\left[\frac{1}{8}{\left(\nabla U_{A}\right)}^{2}+\frac{1}{8}{\left(\nabla U_{B}\right)}^{2}-\frac{1}{4}\nabla^{2}\Delta U\right]+\ldots \end{array}$$

Similarly, $d_{1}$ diagram’s contribution to the concentration variation A can be expressed as

(14) $$\begin{array}{ccc} & & \Delta \phi_{A}\left(r;P_{d_{1}},\Delta \tau \right)=\\ & & k_{0}e^{\mu_{B}}\Delta \tau +Dk_{0}e^{\mu_{B}}\Delta \tau^{2}[\frac{1}{8}{\left(\nabla U_{A}\right)}^{2}+\frac{1}{8}{\left(\nabla U_{B}\right)}^{2}+\frac{1}{4}\nabla^{2}\Delta U+{\left(\nabla \mu_{B}\right)}^{2}+\nabla^{2}\mu_{B}\\ & & +\frac{1}{2}\nabla \mu_{B}\cdot \nabla \Delta U]+\ldots \end{array}$$

The net contribution of $b_{1}$ and $d_{1}$ diagrams to A’s variation can be, thus, obtained as

(15) $$\begin{array}{ccc} & & \Delta \phi_{A}\left(r;P_{d_{1}}+P_{b_{1}},\Delta \tau \right)=\\ & & k_{0}\left(e^{\mu_{B}}-e^{\mu_{A}}\right)\Delta \tau +Dk_{0}\left(e^{\mu_{B}}-e^{\mu_{A}}\right)\Delta \tau^{2}\left[\frac{1}{8}{\left(\nabla U_{A}\right)}^{2}+\frac{1}{8}{\left(\nabla U_{B}\right)}^{2}+\frac{1}{4}\nabla^{2}\Delta U\right]\\ & & +Dk_{0}e^{\mu_{B}}\Delta \tau^{2}\left[\nabla^{2}\mu_{B}+{\left(\nabla \mu_{B}\right)}^{2}+\frac{1}{2}\nabla \mu_{B}\cdot \nabla \Delta U\right]+\ldots \end{array}$$

Similarly, the net contribution of the $b_{1}$ and $d_{1}$ diagrams to B’s variation is

(16) $$\begin{array}{ccc} & & \Delta \phi_{B}\left(r;P_{d_{1}}+P_{b_{1}},\Delta \tau \right)=\\ & & k_{0}\left(e^{\mu_{A}}-e^{\mu_{B}}\right)\Delta \tau +Dk_{0}\left(e^{\mu_{A}}-e^{\mu_{B}}\right)\Delta \tau^{2}\left[\frac{1}{8}{\left(\nabla U_{A}\right)}^{2}+\frac{1}{8}{\left(\nabla U_{B}\right)}^{2}+\frac{1}{4}\nabla^{2}\Delta U\right]\\ & & +Dk_{0}e^{\mu_{A}}\Delta \tau^{2}\left[\nabla^{2}\mu_{A}+{\left(\nabla \mu_{A}\right)}^{2}-\frac{1}{2}\nabla \mu_{A}\cdot \nabla \Delta U\right]+\ldots \end{array}$$

The contributions of other higher-order asymmetric diagrams ($b_{i}$ and $d_{i}$) can be calculated accordingly.

### 5.2. Symmetric Diagrams’ Contributions

Obviously, the probabilities of $a_{1}$ and $c_{1}$ diagrams in Figure 1a,c are just $P_{A}$ (Equation (2)) and $P_{B}$ (Equation (3)), respectively. The contribution of $a_{1}$ to density variation is as follows:

$$\begin{array}{ccc} \Delta \phi_{A}\left(r;P_{a_{1}}^{F},\Delta \tau \right) & & =-\int d\Delta r\phi_{A}\left(r\right)P_{A}\left(r,r+\Delta r;\Delta \tau \right) \end{array}$$

(17) $$\begin{array}{ccc} & & \approx -\phi_{A}-D\Delta \tau \phi_{A}e^{\frac{1}{2}U_{A}}\nabla^{2}e^{-\frac{1}{2}U_{A}}-\frac{1}{2}D^{2}\Delta \tau^{2}\phi_{A}e^{\frac{1}{2}U_{A}}\nabla^{4}e^{-\frac{1}{2}U_{A}},\\ \Delta \phi_{A}\left(r;P_{a_{1}}^{B},\Delta \tau \right) & & =\int d\Delta r\phi_{A}\left(r+\Delta r\right)P_{A}\left(r+\Delta r,r;\Delta \tau \right) \end{array}$$

(18) $$\begin{array}{ccc} & & \approx \phi_{A}+D\Delta \tau e^{-\frac{1}{2}U_{A}}\nabla^{2}\left(\phi_{A}e^{\frac{1}{2}U_{A}}\right)+\frac{1}{2}D^{2}\Delta \tau^{2}e^{-\frac{1}{2}U_{A}}\nabla^{4}\left(\phi_{A}e^{\frac{1}{2}U_{A}}\right), \end{array}$$

where the superscript F in $P_{a_{1}}^{F}$ indicates the forward trajectory, while B indicates the backward trajectory. It can be seen that even though the leading-order contribution is of $\Delta \tau^{0}$, the forward and backward zeroth-order contributions will exactly cancel each other, leaving terms of the first, second and other higher-order terms. These two equations are easily derived by applying Equation (A3).

By adding up Equations (17) and (18), we obtain the total contribution of the forward and backward trajectories of the $a_{1}$ diagram (Figure 1a) as

(19) $$\begin{array}{ccc} & & \Delta \phi_{A}\left(r;P_{a_{1}},\Delta \tau \right)=\Delta \phi_{A}\left(r;P_{a_{1}}^{F},\Delta \tau \right)+\Delta \phi_{A}\left(r;P_{a_{1}}^{B},\Delta \tau \right)\\ & & \approx D\Delta \tau \nabla \left[\phi_{A}\nabla \mu_{A}\right]+\frac{1}{2}D^{2}\Delta \tau^{2}\left\{e^{-\frac{1}{2}U_{A}}\nabla^{4}\left[\phi_{A}e^{\frac{1}{2}U_{A}}\right]-\phi_{A}e^{\frac{1}{2}U_{A}}\nabla^{4}e^{-\frac{1}{2}U_{A}}\right\}, \end{array}$$

where $\mu_{A}=U_{A}+\epsilon_{A}+\ln\phi_{A}$ with $\epsilon_{A}$ being independent of r. Detailed derivation of the first-order term is referred to in Appendix D. Note that the first-order term will obviously reproduce the Smoluchowski equation [20,21] and when $U_{A}=0$, it is reduced to the familiar expression $D\Delta \tau \nabla^{2}\phi_{A}$ encoding Fick’s law.

Similarly, the expressions for $\Delta \phi_{B}\left(r;P_{c_{1}}^{F},\Delta \tau \right)$, $\Delta \phi_{B}\left(r;P_{c_{1}}^{B},\Delta \tau \right)$ and $\Delta \phi_{B}\left(r;P_{c_{1}},\Delta \tau \right)$ can be also obtained as

(20) $$\begin{array}{ccc} \Delta \phi_{B}\left(r;P_{c_{1}}^{F},\Delta \tau \right) & \approx & -\phi_{B}-D\Delta \tau \phi_{B}e^{\frac{1}{2}U_{B}}\nabla^{2}e^{-\frac{1}{2}U_{B}}-\frac{1}{2}D^{2}\Delta \tau^{2}\phi_{B}e^{\frac{1}{2}U_{B}}\nabla^{4}e^{-\frac{1}{2}U_{B}}, \end{array}$$

(21) $$\begin{array}{ccc} \Delta \phi_{B}\left(r;P_{c_{1}}^{B},\Delta \tau \right) & \approx & \phi_{B}+D\Delta \tau e^{-\frac{1}{2}U_{B}}\nabla^{2}\left(\phi_{B}e^{\frac{1}{2}U_{B}}\right)+\frac{1}{2}D^{2}\Delta \tau^{2}e^{-\frac{1}{2}U_{B}}\nabla^{4}\left[\phi_{B}e^{\frac{1}{2}U_{B}}\right], \end{array}$$

(22) $$\begin{array}{ccc} \Delta \phi_{B}\left(r;P_{c_{1}},\Delta \tau \right) & \approx & D\Delta \tau \nabla (\phi_{B}\nabla \mu_{B})\\ & & +\frac{1}{2}D^{2}\Delta \tau^{2}\left\{e^{-\frac{1}{2}U_{B}}\nabla^{4}\left(\phi_{B}e^{\frac{1}{2}U_{B}}\right)-\phi_{B}e^{\frac{1}{2}U_{B}}\nabla^{4}e^{-\frac{1}{2}U_{B}}\right\}. \end{array}$$

The contributions of other higher-order symmetric diagrams ($a_{i}$ and $c_{i}$) can be calculated accordingly.

### 5.3. Summary of DRD Diagram in a Table

Here we summarize the properties of the DRD diagrams of Figure 1 in Table 1.

## 6. Dynamical Equation of Chemical Reactions Coupling with Diffusions

With the results of the above section, dynamical equations can now be derived as follows:

(23) $$\begin{array}{ccc} & & \frac{\partial \phi_{A}}{\partial t}=\frac{1}{\Delta \tau }\sum_{i}\left[\Delta \phi_{A}\left(r;P_{a_{i}},\Delta \tau \right)+\Delta \phi_{A}\left(r;P_{b_{i}},\Delta \tau \right)+\Delta \phi_{A}\left(r;P_{d_{i}},\Delta \tau \right)\right]\\ & & =D\nabla \left(\phi_{A}\nabla \mu_{A}\right)+k_{0}\left(e^{\mu_{B}}-e^{\mu_{A}}\right)+\frac{1}{2}D^{2}\Delta \tau \left[e^{-\frac{1}{2}U_{A}}\nabla^{4}\left(\phi_{A}e^{\frac{1}{2}U_{A}}\right)-\phi_{A}e^{\frac{1}{2}U_{A}}\nabla^{4}e^{-\frac{1}{2}U_{A}}\right]\\ & & +Dk_{0}\left(e^{\mu_{B}}-e^{\mu_{A}}\right)\Delta \tau \left[\frac{1}{8}{\left(\nabla U_{A}\right)}^{2}+\frac{1}{8}{\left(\nabla U_{B}\right)}^{2}+\frac{1}{4}\nabla^{2}\Delta U\right]\\ & & +Dk_{0}e^{\mu_{B}}\Delta \tau \left[\nabla^{2}\mu_{B}+{\left(\nabla \mu_{B}\right)}^{2}+\frac{1}{2}\nabla \mu_{B}\cdot \nabla \Delta U\right]+\ldots \end{array}$$

where $\mu_{A/B}=\epsilon_{A/B}+U_{A/B}+\ln\phi_{A/B}$, $\Delta U=U_{A}-U_{B}$, and $\Delta \tau^{2}$ terms and other higher-order terms are neglected. Note that we set $k_{B}T=1$ through this work for simplicity. Similarly, for B, we have

(24) $$\begin{array}{ccc} & & \frac{\partial \phi_{B}}{\partial t}=D\nabla \left[\phi_{B}\nabla \mu_{B}\right]+k_{0}\left(e^{\mu_{A}}-e^{\mu_{B}}\right)+\frac{1}{2}D^{2}\Delta \tau \left\{e^{-\frac{1}{2}U_{B}}\nabla^{4}\left[\phi_{B}e^{\frac{1}{2}U_{B}}\right]-\phi_{B}e^{\frac{1}{2}U_{B}}\nabla^{4}e^{-\frac{1}{2}U_{B}}\right\}\\ & & +Dk_{0}\left(e^{\mu_{A}}-e^{\mu_{B}}\right)\Delta \tau \left[\frac{1}{8}{\left(\nabla U_{A}\right)}^{2}+\frac{1}{8}{\left(\nabla U_{B}\right)}^{2}+\frac{1}{4}\nabla^{2}\Delta U\right]\\ & & +Dk_{0}e^{\mu_{A}}\Delta \tau \left[\nabla^{2}\mu_{A}+{\left(\nabla \mu_{A}\right)}^{2}-\frac{1}{2}\nabla \mu_{A}\cdot \nabla \Delta U\right]+\ldots \end{array}$$

The above two equations should be the most accurate equations to date that can be derived to describe the dynamics of chemical reaction coupling with diffusions.

First, we would like to analyze the origins of the terms on the right-hand side of the above equations. (i) Even though there are no gradient terms in the leading-order term of the diffusion diagram (e.g., Equation (17) or Figure 1a), the leading-order term of the diffusion diagram and that of its corresponding backward diagram will always cancel each other, leaving terms that have gradient terms. (ii) At the same time, the leading-order term of the reaction diagram and that of its dual diagram will not cancel each other. So the main reaction term in the final dynamical equation has no gradient term. (iii) It is interesting to find that there is a first-order pure diffusion term proportional to $D^{2}\Delta \tau$, which is actually produced by diagrams $a_{1}$ and $c_{1}$, while there is no such first-order pure reaction term proportional to $k_{0}^{2}\Delta \tau$, which can be only produced in the leading-order term in diagrams $a_{2}$ and $c_{2}$ but will be canceled by their backward diagrams. (iv) The left first-order terms in the dynamical equations are the true coupling terms between the diffusion and the reaction that are proportional to $Dk_{0}\Delta \tau$, which are produced by the diagrams $b_{1}$ and $d_{1}$.

Therefore, we have shown that even for monomolecular reactions, there will be reaction-diffusion coupling terms. The reason for this coupling might be that the reaction rate depends on the state of the reactant (e.g., $\propto e^{U_{A}\left(r\right)}$). However, on the mesoscopic scale, this dependence should be replaced with a more precise one that is obtained by averaging over a small volume, and it is like $\propto k_{0}e^{\langle U_{A}\left(r\right)\rangle }$ with $\langle U_{A}\left(r\right)\rangle \approx U_{A}\left(r\right)+a^{\prime }{\left(\Delta r\right)}^{2}{\left(\nabla U_{A}\right)}^{2}+$$b^{\prime }{\left(\Delta r\right)}^{2}\nabla^{2}U_{A}+\ldots =$$U_{A}\left(r\right)+a{\left(\nabla U_{A}\right)}^{2}D\Delta \tau +$$b\nabla^{2}U_{A}D\Delta \tau +\ldots$. The terms $a{\left(\nabla U_{A}\right)}^{2}D\Delta \tau$ and $b\nabla^{2}U_{A}D\Delta \tau$ will finally lead to the reaction–diffusion coupling.

There are two circumstances for which the first-order terms of Δτ cannot be neglected. Here, Δτ should be at least several $\tau_{f}$’s, with $\tau_{f}$ being the mean free time of A/B molecules such that A/B molecules obey Langevin dynamics during Δτ.

First, in the mesoscopic world, the time scale of interest will be close to Δτ, then the first-order terms cannot be neglected, and it would be interesting to investigate the effect of the coupling terms $∼Dk_{0}\Delta \tau$ on the dynamics. We anticipate that these two equations will be useful in the future when researchers are simulating the chemical reactions coupling with diffusions inside the cell, which can be seen as a mesoscopic system.

Second, during the polymerization, polymers or reacting monomers have to diffuse a longer distance than the small-molecule reactions in order to initiate another chemical reaction, which will, in turn, make Δτ larger such that the first-order terms are not negligible, even when viewed from the macroscopic world.

On the contrary, for a macroscopic system composed of small molecules, Δτ is extremely small compared to the time scale of interest. Thus, the first-order terms can be neglected, and the above two equations are reduced to

(25) $$\begin{array}{ccc} & & \frac{\partial \phi_{A}}{\partial t}=D\nabla \left[\phi_{A}\nabla \mu_{A}\right]+k_{0}\left(e^{\mu_{B}}-e^{\mu_{A}}\right), \end{array}$$

(26) $$\begin{array}{ccc} & & \frac{\partial \phi_{B}}{\partial t}=D\nabla \left[\phi_{B}\nabla \mu_{B}\right]+k_{0}\left(e^{\mu_{A}}-e^{\mu_{B}}\right). \end{array}$$

Now, the diffusion and the reaction are actually decoupled since there are no terms $∼Dk_{0}$.

Here are some comments about these two equations:

(i) When there are no chemical reactions, the above equations are actually Smoluchowski equations [20], and these two equations cannot guarantee the local incompressibility of the total concentration since we have not considered this constraint in this work; this will be specially discussed in our future work.
(ii) When the intermolecular interaction is neglected or $U_{A/B}=0$, the above two equations can be further reduced to the traditional reaction–diffusion equations [1,2,3,4,5], which validates the derivation of this work in some sense. Here, we present the traditional reaction–diffusion equations as follows:
  (27) $$\begin{array}{ccc} & & \frac{\partial \phi_{A}}{\partial t}=D\nabla^{2}\phi_{A}+k^{-}\phi_{B}-k^{+}\phi_{A}, \end{array}$$
  (28) $$\begin{array}{ccc} & & \frac{\partial \phi_{B}}{\partial t}=D\nabla^{2}\phi_{B}+k^{+}\phi_{A}-k^{-}\phi_{B}. \end{array}$$
  In particular, the diffusion terms $D\nabla [\phi_{A}\nabla \mu_{A}]$ and $D\nabla [\phi_{B}\nabla \mu_{B}]$ are reduced to $D\nabla^{2}\phi_{A/B}$ ($D\nabla \left[\phi_{A}\nabla \mu_{A}\right]=D\nabla \left[\phi_{A}\nabla \left(\ln\phi_{A}+\epsilon_{A}\right)\right]=D\nabla \left[\phi_{A}\times \phi_{A}^{-1}\nabla \phi_{A}\right]=D\nabla^{2}\phi_{A}$). At the same time, the chemical reaction terms are also reduced to the familiar ones, i.e., $k_{0}\left(e^{\mu_{B}}-e^{\mu_{A}}\right)=k_{0}\left(e^{\ln\phi_{B}+\epsilon_{B}}-e^{\ln\phi_{A}+\epsilon_{A}}\right)=k^{-}\phi_{B}-k^{+}\phi_{A}$ (note that $k^{\pm }=k_{0}e^{\epsilon_{A/B}}$).
(iii) The derived reaction term in this work $k_{0}\left(e^{\mu_{B}}-e^{\mu_{A}}\right)=k^{-}\phi_{B}e^{U_{B}}-k^{+}\phi_{A}e^{U_{A}}$, which is strongly recommended by Carati and Lefever [22], behaves well when $\phi_{A}$ or $\phi_{B}$ or both are close to zero. For example, if $\phi_{A}=10^{-1000000}$ and $\phi_{B}=10^{-1000}$, the reaction rate given by our dynamical equation is obviously very close to zero, but the reaction rate [23,24] given by the term, like $k_{0}\left(\mu_{B}-\mu_{A}\right)$, can be extremely big. The term $k_{0}\left(\mu_{B}-\mu_{A}\right)$ has been used by some previous works [23,24].

## 7. Comparison with Previous Work

To show the difference between our newly derived dynamical equations (Equations (23) and (24)) and the other theory, we give a simulation example. Consider an A/B binary system. The two components can transform into each other through chemical reactions A⇌B, and the interaction between the two components can be expressed as $F_{\mathrm{int}}=\int dr[\chi \phi_{A}\phi_{B}+\frac{1}{2}b_{A}{\left|\nabla \phi_{A}\right|}^{2}$$+\frac{1}{2}b_{B}|\nabla \phi_{B}{|}^{2}]$ with χ the dimensionless interaction parameter and $b_{A/B}$ the interfacial energy coefficients and $U_{A/B}=\delta F_{\mathrm{int}}/\delta \phi_{A/B}$.

**Figure 3:**
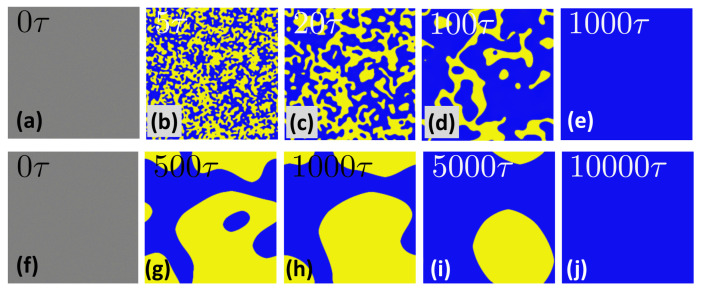
Snapshots of the morphologies of the A/B reacting system at different simulation time steps. The upper snapshots are obtained by Equations (23) and (24) of this work at (**a**) t=0τ, (**b**) t=5τ, (**c**) t=20τ, (**d**) t=100τ and (**e**) t=1000τ, while the bottom snapshots are obtained by the dynamical equations of previous works [23,24] at (**f**) t=0τ, (**g**) t=500τ, (**h**) t=1000τ, (**i**) t=5000τ and (**j**) t=10000τ. The numerical simulations are performed on a 256×256 lattice. $\phi_{A}\left(t=0\right)=1-\phi_{B}\left(t=0\right)\approx 0.5$. D=1.0, $k_{0}=1.0$, χ=3.0, $b_{A}=0.5$, $b_{B}=0.8$ and Δτ=0.05. Note that this system will always reach a uniform equilibrium state as it was pointed out by Carati and Lefever [22].

**Figure 4:**
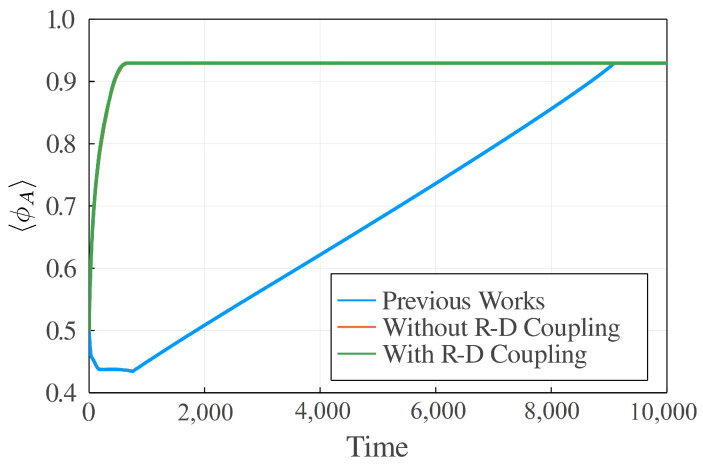
$\langle \phi_{A}\rangle ∼t$ curves obtained by the equations of previous works [23,24], Equations (23) and (24) (without reaction-diffussion (RD) coupling terms) and Equations (25) and (26) (with RD coupling), respectively. The numerical simulations are performed on a 256×256 lattice. D=1.0,$k_{0}=1.0$, χ=3.0, $b_{A}=0.5$, $b_{B}=0.8$ and Δτ=0.05.

**Figure 5:**
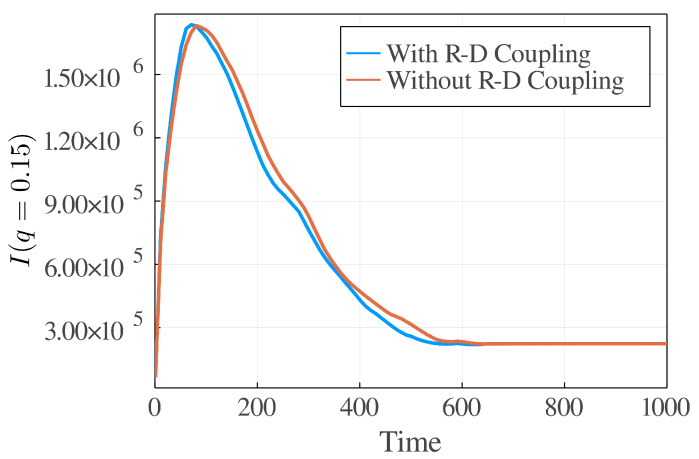
Comparision between the dynamic equations with (Equations (23) and (24)) and without (Equations (25) and (26)) reaction–diffusion coupling terms by showing their light scattering functions at different time steps at a fixed *q*. The numerical simulation is performed on a 256×256 lattice. D=1.0, $k_{0}=1.0$, χ=3.0, $b_{A}=0.5$, $b_{B}=0.8$ and Δτ=0.05. The light intensity at q=0.15 is evaluated by $I\left(q\right)=\;\int \int \;dr_{1}dr_{2}\phi_{A}\left(r_{1}\right)\phi_{A}\left(r_{2}\right)\sin q|r_{1}-r_{2}\left|/q\right|r_{1}-r_{2}|$.

By numerically solving the corresponding dynamical equations with the reaction–diffusion (RD) coupling terms (Equations (23) and (24)), the equations without RD terms (Equations (25) and (26)) and the equations proposed by previous works [23,24], respectively, we see the difference. First, we compared the newly derived dynamic equations in this work with the $k_{0}\left(\mu_{A}-\mu_{B}\right)$-type equations proposed in previous works [23,24] and the difference is significant. The $k_{0}\left(\mu_{A}-\mu_{B}\right)$-type equation is much slower than the newly derived dynamic equations (see Figure 3 and Figure 4) to reach the equilibrium state. We found that $k_{0}\left(\mu_{A}-\mu_{B}\right)$-type equations are numerically instable when the density is extremely small, as mentioned in the preceding section. There is no such problem for our theory. Second, we examined the effect of RD coupling terms (or Δτ terms) in the dynamic equations. By comparing the light scattering results of with (Equations (23) and (24)) and without (Equations (25) and (26)) coupling terms, we can easily find that the dynamics with coupling terms will reach the equilibrium state slightly faster (see Figure 5). In our simulation, we set Δτ=0.05, which is not a big number, but the difference is apparent. Therefore, on the mesoscopic scale, the coupling effect between the reaction and the diffusion might be observable.

## 8. Discussion

Compared with previous works, the newly proposed method and the derived reaction–diffusion equations of this work have the following important features.

(i) The derived dynamical equations in this work contain higher-order terms in Δτ, which show that reactions and diffusions are no longer independent but are coupled.
(ii) The newly derived reaction–diffusion equations are more suitable for the description of reaction–diffusion processes on the mesoscopic scale because the time scale of interest on the mesoscopic scale is comparable to the time scale Δτ. It is also suitable for the description of polymerizations since polymers diffuse much more slowly in polymerization than the small-molecule diffusion, which might make the reaction–diffusion coupling effect observable on the macroscopic scale. Nevertheless, the above anticipations still need to be validated by more simulations or experiments in the future.
(iii) The newly proposed method helps us derive a more consistent reaction–diffusion equation directly from the microscopic dynamics. The reaction term in the newly derived equation is more reasonable than the previous one, especially for the extremely small density region.
(iv) Physical origins of every term in the derived dynamical equations can be clearly seen in the DRD diagrams. For example, one can clearly see that the reason for the diffusion terms containing no pure local terms but gradients is because even though the leading-order term of a pure diffusion diagram (e.g., $a_{1}$ in Figure 1a) is purely local, it will be exactly canceled by the leading-order term of its backward diagram, leaving next- and higher-order terms, which are all non-pure local terms. On the contrary, the reaction term can be purely local, which is because the leading order term of the reaction diagram cannot be canceled by that of its dual diagram. Actually, the above results are also in accord with Curie’s principle about the symmetry of cause and effect.

However, improvements can be also made on the present work in the future.

(i) In the derivation, the definition of density is actually not strict and accurate. A more accurate definition of the density should depend on the way of how we define the density or depend on the box size which we use to define the local density. In our future work, we will try to introduce the more accurate definition of density that has been employed by other works [17,18].
(ii) In this work, the binary reversible reaction is actually very special in the sense that it involves no explicit intermolecular collisions, which is normally impossible for other reactions. In our next work, we will consider more complicated reactions involving more than two types of reacting molecules and explicitly consider intermolecular collisions in theory. Note that to include collisions, the new definition of the density mentioned in (i) will play an important role.
(iii) In this work, we have not considered the local incompressibility of the total density. In our future work, we will specially discuss how the local incompressibility spontaneously arises from the microscopic world without using any penalty energy to impose the incompressibility.
(iv) In the future, particle-based simulations are still required to be done to validate the higher-order terms in the reaction–diffusion equations derived in this work.

## 9. Conclusions

In this work, we proposed a Feynman path integral-like method to derive the reaction–diffusion equations that are suitable for the description of the reaction–diffusion dynamics on the mesoscopic scale, where, different from those on the macroscopic scale, reactions and diffusions might deeply couple. By noting that a previous simple physical process might consist of a series of diffusion–reaction–diffusion trajectories or diagrams, the new method was able to derive the reaction–diffusion equation for the A/B reacting system of very high accuracy. The higher-order terms in the newly derived equations show that reactions and diffusions might couple, even for monomolecular reactions, and become manifested on the mesoscopic scale. The newly derived equations are reduced to the traditional reaction–diffusion equations when the intermolecular interactions and higher-order terms are both neglected. The proposed Feynman path integral-like method can be also extended to polymeric systems to derive more accurate equations for the description of polymerization dynamics.

## Figures and Tables

**Table 1:** Summary of diffusion–reaction–diffusion (DRD) diagrams of Figure 1.

DRD Diagram	Name	Order (in Δτ)	Symmetry	Probability	Contribution to Δϕ	Dual Diagram
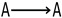	$a_{1}$	0th	Symmetric	Equation (2)	Equations (17)–(19)	$a_{1}$
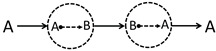	$a_{2}$	2nd	Symmetric	Equation (8)	/	$a_{2}$
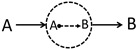	$b_{1}$	1st	Asymmetric	Equation (6)	Equations (11) and (12)	$d_{1}$
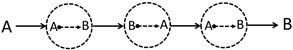	$b_{2}$	3rd	Asymmetric	/	/	$d_{2}$
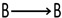	$c_{1}$	0th	Symmetric	Equation (3)	Equations ((20)–(22)	$c_{1}$
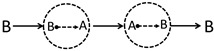	$c_{2}$	2nd	Symmetric	/	/	$c_{2}$
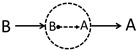	$d_{1}$	1st	Asymmetric	Equation (7)	Equations (13) and (14)	$b_{1}$
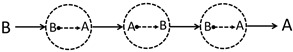	$d_{2}$	3rd	Asymmetric	/	/	$b_{2}$

## Data Availability

Not applicable.

## Appendix A. Useful Formulae

We first list a series of useful formulae here. The first one evaluates the variations of Δr for a particle that obeys the Langevin dynamics:

(A1) $$\begin{array}{c} \langle \Delta r^{2}\rangle =\int d\Delta r{\left(\frac{1}{4\pi D\Delta \tau }\right)}^{\frac{3}{2}}\Delta r^{2}\exp\left\{-\frac{\Delta r^{2}}{4D\Delta \tau }\right\}=6D\Delta \tau , \end{array}$$

where *D* is the diffusion coefficient. Note that $\langle \Delta x^{2}\rangle =\langle \Delta y^{2}\rangle =\langle \Delta z^{2}\rangle =2D\Delta \tau$.

The second one is useful for connecting two joint trajectories and it is as follows:

(A2) $$\begin{array}{ccc} & & {\left(\frac{1}{4D\pi \tau_{1}}\right)}^{\frac{3}{2}}{\left(\frac{1}{4D\pi \tau_{2}}\right)}^{\frac{3}{2}}\int \exp\left\{-\frac{\Delta r_{1}^{2}}{4D\tau_{1}}-\frac{{\left(\Delta r-\Delta r_{1}\right)}^{2}}{4D\tau_{2}}\right\}d\Delta r_{1}\\ & = & {\left(\frac{1}{4D\pi \tau_{1}}\right)}^{\frac{3}{2}}{\left(\frac{1}{4D\pi \tau_{2}}\right)}^{\frac{3}{2}}\int \exp\left\{-\frac{\tau_{1}+\tau_{2}}{4D\tau_{1}\tau_{2}}{\left(\Delta r_{1}-\frac{\tau_{1}}{\tau_{1}+\tau_{2}}\Delta r\right)}^{2}-\frac{\Delta r^{2}}{4D(\tau_{1}+\tau_{2})}\right\}d\Delta r_{1}\\ & = & {\left(\frac{1}{4D\pi (\tau_{1}+\tau_{2})}\right)}^{\frac{3}{2}}\exp\left\{-\frac{\Delta r^{2}}{4D(\tau_{1}+\tau_{2})}\right\}. \end{array}$$

The third one evaluates the average of a function f(r+Δr), which will frequently be used in this work:

(A3) $$\begin{array}{c} \int d\Delta r{\left(\frac{1}{4\pi D\Delta \tau }\right)}^{\frac{3}{2}}f\left(r+\Delta r\right)\exp\left\{-\frac{\Delta r^{2}}{4D\Delta \tau }\right\}=\exp\left\{D\Delta \tau \nabla^{2}\right\}f\left(r\right). \end{array}$$

This identity can be proved in the Fourier space by replacing f(r+Δr) with $\int dkf\left(k\right)e^{ikr+ik\Delta r}$ as follows:

$$\begin{array}{ccc} & & \int d\Delta r{\left(\frac{1}{4\pi D\Delta \tau }\right)}^{\frac{3}{2}}f\left(r+\Delta r\right)\exp\left\{-\frac{\Delta r^{2}}{4D\Delta \tau }\right\}\\ & & =\int d\Delta rdk{\left(\frac{1}{4\pi D\Delta \tau }\right)}^{\frac{3}{2}}f\left(k\right)\exp\left\{ikr\right\}\exp\left\{-\frac{\Delta r^{2}}{4D\Delta \tau }+ik\Delta r\right\}\\ & & =\int dk\exp\left\{-D\Delta \tau k^{2}\right\}f\left(k\right)\exp\left\{ikr\right\}\\ & & =\exp\left\{D\Delta \tau \nabla^{2}\right\}f\left(r\right). \end{array}$$

The fourth one can be used to directly evaluate the diagrams in Figure 1 and it is as follows:

$$\begin{array}{ccc} & & \int d\Delta r^{\prime }\int_{0}^{\tau }d\tau^{\prime }{\left(\frac{1}{4D\pi \tau^{\prime }}\right)}^{\frac{3}{2}}{\left(\frac{1}{4D\pi (\tau -\tau^{\prime })}\right)}^{\frac{3}{2}}g\left(r+\Delta r^{\prime }\right)\exp\left\{-\frac{\Delta r^{\prime 2}}{4D\tau^{\prime }}-\frac{{\left(\Delta r-\Delta r^{\prime }\right)}^{2}}{4D(\tau -\tau^{\prime })}\right\} \end{array}$$

(A4) $$\begin{array}{ccc} & & ={\left(\frac{1}{4D\pi \tau }\right)}^{\frac{3}{2}}\exp\left\{-\frac{\Delta r^{2}}{4D\tau }\right\}\int_{0}^{\tau }d\tau^{\prime }e^{\frac{D\tau^{\prime }\left(\tau -\tau^{\prime }\right)}{\tau }\nabla^{2}}g\left(r+\frac{\tau^{\prime }}{\tau }\Delta r\right) \end{array}$$

(A5) $$\begin{array}{ccc} & & ={\left(\frac{1}{4D\pi \tau }\right)}^{\frac{3}{2}}\exp\left\{-\frac{\Delta r^{2}}{4D\tau }\right\}\left[g\tau +\frac{1}{2}\nabla g\cdot \Delta r\tau +\frac{1}{6}\partial_{\alpha }\partial_{\beta }g\Delta x^{\alpha }\Delta x^{\beta }\tau +\frac{1}{6}D\nabla^{2}g\cdot \tau^{2}+\ldots \right]. \end{array}$$

Note that in the above equation, we specially use the Einstein summation $\partial_{\alpha }\partial_{\beta }g\Delta x^{\alpha }\Delta x^{\beta }=\partial_{x}^{2}g\Delta x^{2}+\partial_{y}^{2}g\Delta y^{2}+\partial_{z}^{2}g\Delta z^{2}+2\partial_{x}\partial_{y}g\Delta x\Delta y+2\partial_{y}\partial_{z}g\Delta y\Delta z+2\partial_{z}\partial_{x}g\Delta z\Delta x$ while $\nabla^{2}g\Delta r^{2}=\partial_{\alpha }\partial^{\alpha }g\Delta x^{\beta }\Delta x_{\beta }=\left(\partial_{x}^{2}g+\partial_{y}^{2}g+\partial_{z}^{2}g\right)\left(\Delta x^{2}+\Delta y^{2}+\Delta z^{2}\right)$.

Equation (A4) can be proved as follows, by plugging $g(r+\Delta r^{\prime })$ into Equation (A2):

$$\begin{array}{ccc} & & \int d\Delta r\int_{0}^{\tau }d\tau^{\prime }{\left(\frac{1}{4D\pi \tau^{\prime }}\right)}^{\frac{3}{2}}{\left(\frac{1}{4D\pi (\tau -\tau^{\prime })}\right)}^{\frac{3}{2}}g\left(r+\Delta r^{\prime }\right)\exp\left\{-\frac{\Delta r^{\prime 2}}{4D\tau^{\prime }}-\frac{{\left(\Delta r-\Delta r^{\prime }\right)}^{2}}{4D(\tau -\tau^{\prime })}\right\}\\ & & =\int d\Delta r\int_{0}^{\tau }d\tau^{\prime }{\left(\frac{1}{4D\pi \tau^{\prime }}\right)}^{\frac{3}{2}}{\left(\frac{1}{4D\pi (\tau -\tau^{\prime })}\right)}^{\frac{3}{2}}\exp\left\{-\frac{{\left(\Delta r^{\prime }-\frac{\tau^{\prime }}{\tau }\Delta r\right)}^{2}\tau }{4D\tau^{\prime }\left(\tau -\tau^{\prime }\right)}-\frac{\Delta r^{2}}{4D\tau }\right\}g\left(r+\Delta r^{\prime }\right)\\ & & ={\left(\frac{1}{4D\pi \tau }\right)}^{\frac{3}{2}}\exp\left\{-\frac{\Delta r^{2}}{4D\tau }\right\}\int d\Delta r^{\prime }\int_{0}^{\tau }d\tau^{\prime }{\left(\frac{\tau }{4D\pi \left(\tau -\tau^{\prime }\right)\tau^{\prime }}\right)}^{\frac{3}{2}}\\ & & \times \exp\left\{-\frac{{\left(\Delta r^{\prime }-\frac{\tau^{\prime }}{\tau }\Delta r\right)}^{2}\tau }{4D\tau^{\prime }\left(\tau -\tau^{\prime }\right)}\right\}g\left(r+\Delta r^{\prime }\right)\\ & & ={\left(\frac{1}{4D\pi \tau }\right)}^{\frac{3}{2}}\exp\left\{-\frac{\Delta r^{2}}{4D\tau }\right\}\int_{0}^{\tau }d\tau^{\prime }e^{\frac{D\tau^{\prime }\left(\tau -\tau^{\prime }\right)}{\tau }\nabla^{2}}g\left(r+\frac{\tau^{\prime }}{\tau }\Delta r\right), \end{array}$$

where in the last equality the identity Equation (A3) is applied.

## Appendix B. Probability of a Trajectory

To obtain the probability of a trajectory from r to r+Δr, it is sufficient to only consider the 1D case. The extension to 3D is straightforward.

If the particle obeys the following Langevin dynamics,

$$\begin{array}{ccc} \frac{\partial x}{\partial t} & = & \mu F(x)+\xi , \end{array}$$

where $\langle \xi \left(t\right)\xi \left(t^{\prime }\right)\rangle =2D\delta \left(t-t^{\prime }\right)$ and D=μT, then the probability of a trajectory x(t) with 0<t<Δτ is,

(A6) $$\begin{array}{c} P\left[x\left(t\right)\right]\propto \exp\left\{-\int_{0}^{\Delta \tau }dt\left[\frac{{\left(\dot{x}-\mu F\right)}^{2}}{4D}+\frac{\mu \partial_{x}F}{2}\right]\right\}, \end{array}$$

according to the fluctuation theorem [25,26].

If *F* is the gradient of some potential *U* or conservative, then the probability P(Δx;Δτ) can be obtained as, by path integrating P[x(t)] over x(t),

$$\begin{array}{ccc} & & P(\Delta x;\Delta \tau )=\int P[x(t)]Dx(t)\\ & & \propto \lim_{\Delta t\to 0}{\left(\frac{1}{4\pi D\Delta t}\right)}^{\frac{\Delta \tau }{2\Delta t}}\int \Pi_{k}dx\left(k\right)e^{-\sum_{k=1}^{\frac{\Delta \tau }{\Delta t}}\left[\frac{{\left(x\left(k+1\right)-x\left(k\right)\right)}^{2}}{4D\Delta \tau }+\sum_{k}\frac{\mu \left(x\left(k+1\right)-x\left(k\right)\right)\partial_{x}U}{2D}+\sum_{k}\left[\frac{\mu \partial_{x}F}{2}-\frac{\mu^{2}{\left(\partial_{x}U\right)}^{2}}{4D}\right]\Delta t\right]}\\ & & =e^{\frac{1}{2}\left[U\left(x\right)-U\left(x+\Delta x\right)\right]}\lim_{\Delta t\to 0}{\left(\frac{1}{4\pi D\Delta t}\right)}^{\frac{\Delta \tau }{2\Delta t}}\int \Pi_{k}d\Delta x_{k}e^{-\sum_{k=1}^{\frac{\Delta \tau }{\Delta t}}\left[\frac{\Delta x_{k}^{2}}{4D\Delta t}+\sum_{k}\left[\frac{\mu \partial_{x}F}{2}-\frac{\mu^{2}{\left(\partial_{x}U\right)}^{2}}{4D}\right]\Delta t\right]}\\ & & \approx e^{\frac{1}{2}\left[U\left(x\right)-U\left(x+\Delta x\right)\right]}\lim_{\Delta t\to 0}{\left(\frac{1}{4\pi D\Delta t}\right)}^{\frac{\Delta \tau }{2\Delta t}}\int \Pi_{k}d\Delta x_{k}e^{-\sum_{k=1}^{\frac{\Delta \tau }{\Delta t}}\left[\frac{\Delta x_{k}^{2}}{4D\Delta t}\right]}\\ & & ={\left(\frac{1}{4\pi D\Delta \tau }\right)}^{1/2}e^{-\frac{\Delta x^{2}}{4D\Delta \tau }}e^{\frac{1}{2}\left[U\left(x\right)-U\left(x+\Delta x\right)\right]}, \end{array}$$

where in the last equality, Equation (A2) is applied progressively for Δτ/Δt times.

This result can be easily extended to 3D as follows:

(A7) $$\begin{array}{c} P\left(r,r+\Delta r;\Delta \tau \right)={\left(\frac{1}{4\pi D\Delta \tau }\right)}^{3/2}e^{-\frac{\Delta r^{2}}{4D\Delta \tau }}e^{\frac{1}{2}\left[U\left(r\right)-U\left(r+\Delta r\right)\right]}. \end{array}$$

Note that we have changed P[r(t)] to P(r,r+Δr;Δτ) since now the probability only depends on the two ends and the time span Δτ.

The above equation evaluates the probability of a particle diffusing from r to r+Δr within Δτ (it is assumed that there is already a particle in the position r), while the probability of observing a particle diffusing from r to r+Δr within Δτ should be multiplied by the number density ϕ (or $e^{\ln\phi }$) at r, which can be expressed as follows:

(A8) $$\begin{array}{c} \phi \left(r\right)P\left(r,r+\Delta r;\Delta \tau \right)={\left(\frac{1}{4\pi D\Delta \tau }\right)}^{3/2}e^{-\frac{\Delta r^{2}}{4D\Delta \tau }}e^{\ln\phi \left(r\right)+\frac{1}{2}\left[U\left(r\right)-U\left(r+\Delta r\right)\right]}. \end{array}$$

This expression is in accord with the result obtained in the fluctuation theory [26], where the exponent $-\ln\phi \left(r\right)-\frac{1}{2}\left[U\left(r\right)-U\left(r+\Delta r\right)\right]$ is defined as the second entropy of the transition.

## Appendix C. Derivation of Equation (11)

According to Equation (7), we have

(A9) $$\begin{array}{ccc} & & \Delta \phi_{A}\left(r;P_{b_{1}},\Delta \tau \right)=-\int d\Delta r\phi_{A}\left(r\right)P_{b_{1}}\left(r,r+\Delta r;\Delta \tau \right)\\ & & =-\int d\Delta r\phi_{A}\left(r\right){\left(\frac{1}{4\pi D\Delta \tau }\right)}^{3/2}e^{-\frac{\Delta r^{2}}{4D\Delta \tau }}k^{+}e^{\frac{1}{2}\left[U_{A}-U_{B}\left(r+\Delta r\right)\right]}\\ & & \{e^{\frac{1}{2}\left[U_{A}+U_{B}\right]}\Delta \tau +\frac{1}{2}\left(\nabla e^{\frac{1}{2}\left[U_{A}+U_{B}\right]}\right)\cdot \Delta r\Delta \tau +\frac{1}{6}\left(\partial_{\alpha }\partial_{\beta }e^{\frac{1}{2}\left[U_{A}+U_{B}\right]}\Delta x^{\alpha }\Delta x^{\beta }\Delta \tau +\ldots \right)\}. \end{array}$$

For the first term in the braces, using Equation (A3), we can obtain

(A10) $$\begin{array}{ccc} & & -\int d\Delta r\phi_{A}\left(r\right){\left(\frac{1}{4\pi D\Delta \tau }\right)}^{3/2}e^{-\frac{\Delta r^{2}}{4D\Delta \tau }}k^{+}e^{\frac{1}{2}\left[U_{A}-U_{B}\left(r+\Delta r\right)\right]}\left\{e^{\frac{1}{2}\left[U_{A}+U_{B}\right]}\Delta \tau \right\}\\ & = & -k_{0}e^{\mu_{A}}e^{\frac{1}{2}U_{B}}e^{D\Delta \tau \nabla^{2}}e^{-\frac{1}{2}U_{B}}\Delta \tau \\ & = & -k_{0}e^{\mu_{A}}-k_{0}D\Delta \tau^{2}e^{\mu_{A}}e^{\frac{1}{2}U_{B}}D\nabla^{2}e^{-\frac{1}{2}U_{B}}\\ & = & -k_{0}e_{A}^{\mu }-k_{0}De^{\mu_{A}}\left[\frac{1}{4}{\left(\nabla U_{B}\right)}^{2}-\frac{1}{2}\nabla^{2}U_{B}\right]. \end{array}$$

For the second term in the braces, we obtain

$$\begin{array}{ccc} & & -\int d\Delta r\phi_{A}\left(r\right){\left(\frac{1}{4\pi D\Delta \tau }\right)}^{3/2}e^{-\frac{\Delta r^{2}}{4D\Delta \tau }}k^{+}e^{\frac{1}{2}\left[U_{A}-U_{B}\left(r+\Delta r\right)\right]}\left\{\frac{1}{2}\left(\nabla e^{\frac{1}{2}\left[U_{A}+U_{B}\right]}\right)\cdot \Delta r\Delta \tau \right\}\\ & \approx & -\frac{1}{2}k_{0}e^{\mu_{A}-U_{A}}\Delta \tau \;\partial_{\alpha }e^{-\frac{1}{2}U_{B}}\partial_{\beta }e^{\frac{1}{2}\left[U_{A}+U_{B}\right]}\langle \Delta r^{\alpha }\Delta r^{\beta }\rangle \\ & \approx & -k_{0}D\Delta \tau^{2}e^{\mu_{A}-U_{A}}\;\nabla e^{-\frac{1}{2}U_{B}}\cdot \nabla e^{\frac{1}{2}\left[U_{A}+U_{B}\right]}\\ & \approx & -\frac{k_{0}}{4}D\Delta \tau^{2}e^{\mu_{A}}\left[{\left(\nabla U_{B}\right)}^{2}+\nabla U_{A}\cdot \nabla U_{B}\right]. \end{array}$$

Similarly, for the third term in the braces, we can derive that the average is equal to $-k_{0}D\Delta \tau^{2}e^{\mu_{A}}\left\{\frac{1}{4}\nabla^{2}\left(U_{A}+U_{B}\right)+\frac{1}{8}{\left(\nabla \left(U_{A}+U_{B}\right)\right)}^{2}\right\}$.

Therefore, by summing up the contributions above, we arrive at Equation (11),

(A11) $$\begin{array}{ccc} & & \Delta \phi_{A}\left(r;P_{b_{1}},\Delta \tau \right)=-\int d\Delta r\phi_{A}\left(r\right)P_{b_{1}}\left(r,r+\Delta r;\Delta \tau \right)\\ & & =-k_{0}e^{\mu_{A}}\Delta \tau -Dk_{0}e^{\mu_{A}}\Delta \tau^{2}\left[\frac{1}{8}{\left(\nabla U_{A}\right)}^{2}+\frac{1}{8}{\left(\nabla U_{B}\right)}^{2}+\frac{1}{4}\nabla^{2}\left(U_{A}-U_{B}\right)\right]+\ldots \end{array}$$

## Appendix D. Derivation of the Smoluchowski Term [20] in the Main Text

By noting that $\nabla^{2}e^{\frac{1}{2}U_{A}}=e^{\frac{1}{2}U_{A}}\left[\frac{1}{4}{\left(\nabla U_{A}\right)}^{2}+\frac{1}{2}\nabla^{2}U_{A}\right]$ and $\nabla^{2}e^{-\frac{1}{2}U_{A}}=e^{-\frac{1}{2}U_{A}}\left[\frac{1}{4}{\left(\nabla U_{A}\right)}^{2}-\frac{1}{2}\nabla^{2}U_{A}\right]$, the first-order term (or the Smoluchowski term [20]) in Equation (19) is derived as follows:

$$\begin{array}{ccc} & & D\Delta \tau e^{-\frac{1}{2}U_{A}}\nabla^{2}\left[\phi_{A}e^{\frac{1}{2}U_{A}}\right]-D\Delta \tau e^{\frac{1}{2}U_{A}}\phi_{A}\nabla^{2}e^{-\frac{1}{2}U_{A}}\\ & & =D\Delta \tau [\nabla^{2}\phi_{A}+2e^{-\frac{1}{2}U_{A}}\nabla \phi_{A}\nabla e^{\frac{1}{2}U_{A}}+\phi_{A}e^{-\frac{1}{2}U_{A}}\nabla^{2}e^{\frac{1}{2}U_{A}}-\phi_{A}e^{\frac{1}{2}U_{A}}\nabla^{2}e^{-\frac{1}{2}U_{A}}]\\ & & =D\Delta \tau [\nabla^{2}\phi_{A}+\nabla \phi_{A}\nabla U_{A}+\phi_{A}\left[\frac{1}{4}{\left(\nabla U_{A}\right)}^{2}+\frac{1}{2}\nabla^{2}U_{A}\right]-\phi_{A}\left[\frac{1}{4}{\left(\nabla U_{A}\right)}^{2}-\frac{1}{2}\nabla^{2}U_{A}\right]]\\ & & =D\Delta \tau [\nabla^{2}\phi_{A}+\nabla \phi_{A}\nabla U_{A}+\phi_{A}\nabla^{2}U_{A}]\\ & & =D\Delta \tau \nabla [\phi_{A}\nabla \left(\ln\phi_{A}+U_{A}\right)]\\ & & =D\Delta \tau \nabla [\phi_{A}\nabla \mu_{A}] \end{array}$$

where $\mu_{A}=\ln\phi_{A}+U_{A}+\epsilon_{A}$. Note that $\nabla \epsilon_{A}=0$.
